# Comprehensive genetic exploration of selective tooth agenesis of mandibular incisors by exome sequencing

**DOI:** 10.1038/hgv.2017.5

**Published:** 2017-02-23

**Authors:** Tetsutaro Yamaguchi, Kazuyoshi Hosomichi, Keisuke Yano, Yong-Il Kim, Hirofumi Nakaoka, Ryosuke Kimura, Hirotada Otsuka, Naoko Nonaka, Shugo Haga, Masahiro Takahashi, Tatsuo Shirota, Yoshiaki Kikkawa, Atsushi Yamada, Ryutaro Kamijo, Soo-Byung Park, Masanori Nakamura, Koutaro Maki, Ituro Inoue

**Affiliations:** 1 Department of Orthodontics, School of Dentistry, Showa University, Tokyo, Japan; 2 Division of Human Genetics, National Institute of Genetics, Shizuoka, Japan; 3 Department of Bioinformatics and Genomics, Graduate School of Medical Sciences, Kanazawa University, Ishikawa, Japan; 4 Verde Orthodontic Dental Clinic, Tokyo, Japan; 5 Department of Orthodontics, School of Dentistry, Pusan National University, Busan, South Korea; 6 Department of Human Biology and Anatomy, Graduate School of Medicine, University of the Ryukyus, Okinawa, Japan; 7 Department of Oral Anatomy and Developmental Biology, Showa University School of Dentistry, Tokyo, Japan; 8 Department of Oral and Maxillofacial Surgery, School of Dentistry, Showa University, Tokyo, Japan; 9 Mammalian Genetics Project, Tokyo Metropolitan Institute of Medical Science, Tokyo, Japan; 10 Department of Biochemistry, School of Dentistry, Showa University, Tokyo, Japan

## Abstract

Tooth agenesis is described as the absence of one or more teeth. It is caused by a failure in tooth development and is one of the most common human developmental anomalies. We herein report genomic analyses of selective mandibular incisor agenesis (SMIA) using exome sequencing. Two Japanese families with SMIA were subjected to exome sequencing, and family with sequence similarity 65 member A (*FAM65*), nuclear factor of activated T-cells 3 (*NFATC3*) and cadherin-related 23 gene (*CDH23*) were detected. In the follow-up study, 51 Japanese and 32 Korean sporadic patients with SMIA were subjected to exome analyses, and 18 reported variants in *PAX9*, *AXIN2*, *EDA*, *EDAR*, *WNT10A*, *BMP2* and *GREM2* and 27 variants of *FAM65*, *NFATC3* and *CDH23* were found in 38 patients. Our comprehensive genetic study of SMIA will pave the way for a full understanding of the genetic etiology of SMIA and provide targets for treatment.

## Introduction

Humans usually develop 32 permanent teeth, including third molars. Human teeth are spatially specialized and classified as incisors and canines (the anterior teeth) and premolars and molars (the posterior teeth). Tooth agenesis denotes a pathological condition involving the absence of teeth because of a developmental failure. Selective (nonsyndromic) tooth agenesis is one of the most common dental anomalies; it also accompanies several syndromes, such as van der Woude syndrome, ectodermal dysplasias, oral–facial–digital syndrome type I, Rieger syndrome, holoprosencephaly and cleft lip and palate.^1^

Tooth agenesis most often occurs at the third molars across all populations.^2^ In populations of European descent, the second most common congenital missing teeth are the mandibular second premolars, followed by either the permanent maxillary lateral incisors^3^ or the maxillary second premolars.^4,5^ In Japanese patients and other Asians, the second most common missing teeth are the mandibular second premolars, followed by the mandibular and maxillary lateral incisors, and the maxillary second premolars.^6–8^ A notable difference has been observed in the frequencies of mandibular incisor agenesis between Japanese (1–2%)^7,8^ and European populations (0.2%).^9^

Selective tooth agenesis is known to be associated with variants of msh homeobox 1 (*MSX1*), paired box 9 (*PAX9*), axin 2 (*AXIN2*), ectodysplasin A (*EDA*), ectodysplasin A receptor (*EDAR*), EDAR-associated death domain (*EDARADD*), wingless-type MMTV integration site family member 10A (*WNT10A*),^10^ bone morphogenetic protein 2 (*BMP2*),^11^ gremlin 2 and DAN family BMP antagonist (*GREM2*).^12^ The identification of causality for agenesis of the mandibular incisors, referred to herein as selective mandibular incisor agenesis (SMIA), may enable us to elucidate the precise molecular mechanisms of temporal and spatial gene regulation in the distinctive development of human dentition. Therefore, in the current study, we performed exome analyses on 2 families and 83 sporadic patients of Japanese and Koreans demonstrating possible involvement of family with sequence similarity 65 member A (*FAM65*), nuclear factor of activated T-cells 3 (*NFATC3*) or cadherin-related 23 gene (*CDH23*) in the pathogenesis of SMIA in addition to the reported genes.

## Materials and methods

### Subjects

Patients with SMIA were diagnosed and recruited from the Showa University Dental Hospital (Tokyo, Japan) and affiliated hospitals. Standardized assessments, including panoramic radiographs, dental casts, intraoral photographs and anamnestic data, were performed in all patients. Dental specialists diagnosed the dentition status of all subjects and their available siblings and parents. Patients with developmental anomalies, such as ectodermal dysplasia, cleft lip or palate, or Down’s syndrome or those who had undergone orthodontic treatment previously were excluded from the study. This procedure has been considered reliable for diagnosing anomalies in tooth number in several studies.^13,14^

**Figure 1:**
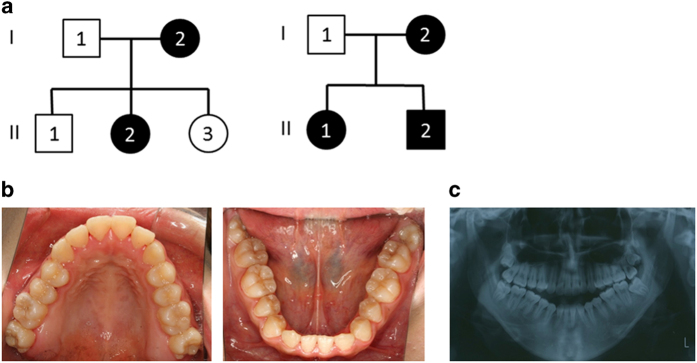
(**a**) Left; Family A, right; Family B. The pedigree showing autosomal-dominant inheritance of selective mandibular incisor agenesis. (**b**) Intraoral photographs and (**c**) panoramic radiograph of the proband (II:1) at 17 years of age. Note the absence of two mandibular incisors.

We recruited two Japanese family (Family A containing two affected and three unaffected individuals; Family B containing three affected and one unaffected individuals) showing a dominant transmission of SMIA (Figure 1). We also recruited 51 Japanese and 32 Korean patients with sporadic SMIA (aged 10–37 years). Twelve patients (SH3, SH14, SH23, SH24, SH31, SH35, SH38, SH49, SH50, SH51, SH66 and SH69) had agenesis of 2 incisors and 71 patients had agenesis of 1 incisor. The possibility of the misdiagnosis of agenesis of the mandibular central and lateral incisors caused by anatomical artifacts derived from the superposition of cervical vertebrae on the mental region should be noted.^8^ Therefore, in the present study, we did not distinguish agenesis between mandibular central and lateral incisors. Family histories of these patients were incomplete. Although the hearing ability of the patients with SMIA was not examined by an otolaryngologist, the dental doctor in charge confirmed that the patients could hold a normal conversation.

We collected saliva specimens from each patient and extracted DNA using the Oragene DNA Kit (DNA Genotek, Ottawa, Canada). This study was conducted under the approval of ethical committees at Showa University, Pusan National University and the National Institute of Genetics and performed according to the ethical principles defined in the Declaration of Helsinki. All subjects gave their informed consent to participate in the study.

### Exome sequencing

Exome sequencing was performed for 5 individuals in Family A, 4 individuals in Family B and 83 sporadic patients (51 Japanese and 32 Koreans). DNA samples (3 μg) were subjected to exome capture using the SureSelect Human All Exon Kit (Agilent Technologies, Santa Clara, CA, USA) according to the manufacturer’s instructions. In brief, genomic DNA was randomly fragmented by sonication under standard conditions (Covaris, Woburn, MA, USA), followed by end repair, the addition of a single A base, adaptor ligation and gel electrophoresis to isolate 300-bp fragments, followed by PCR amplification. The captured DNA underwent high-throughput sequencing using the HiSeq2500 system (Illumina, San Diego, CA, USA).

Next, the size-selected libraries were used for cluster generation on the flow cells. All prepared flow cells were run on the Illumina HiSeq2500 using paired-end 100-bp reads. Reads were mapped to the reference genome (UCSC hg19) using Burrows-Wheeler Aligner v.0.7.9.^15^ Burrows-Wheeler Aligner-generated SAM files were converted to BAM format, then sorted and indexed using SAMtools v.0.1.18.^16^ Duplicated reads were marked with Picard v.1.102 (https://github.com/broadinstitute/picard). The files obtained in BAM format were analyzed using GATK v.2.7 following their best practice guidelines.^17^ In brief, BAM files were first subjected to insertion or deletion (indel) realignment, base quality score recalibration and variant calling with the UnifiedGenotyper walker to obtain potential variants in the Variant Call Format file. These variants were annotated using the algorithm (table.annovar.pl) in ANNOVAR (version 2013jul21).^18^ For gene annotation, we used the RefSeq gene database (build hg19),^19^ while variant annotation was based on dbSNP (dbSNP 137), the 1000 Genomes Project database^20^ and 1208 Japanese data in the Human Genetic Variation database (http://www.genome.med.kyoto-u.ac.jp/SnpDB/index.html).

### Filtering to detect causal variants

In Family A and Family B, variants detected from exome sequencing data were further analyzed by performing three filtering steps based on different criteria. In the first filtering step, we selected missense and nonsense variants, splice-site single-nucleotide variants and coding indels. The second filtering step was based on the frequency in the Human Genetic Variation database. Variants with a frequency <5% in the Human Genetic Variation database were filtered as SMIA candidates. Finally, heterozygous variants co-segregated in the family were selected. After these filtering steps, candidate variants were confirmed for all family members by Sanger sequencing on the 3730xl DNA Analyzer (Life Technologies, Carlsbad, CA, USA). Functional estimation and the conservation score of the variants were evaluated by prediction tools Polymorphism Phenotyping v2^21^ and Genomic Evolutionary Rate Profiling,^22^ respectively.

In the 83 sporadic Japanese and Korean patients (SH1-SH83), variants of *PAX9*, *AXIN2*, *EDA*, *EDAR*, *WNT10A*, *BMP2* and *GREM2* previously reported in selective tooth agenesis were identified using exome data. Functional estimation and the conservation score of the variants were evaluated as before by Polymorphism Phenotyping v2 and Genomic Evolutionary Rate Profiling.

## Results

### Variants detection in families with exome sequencing

Exome sequencing was performed on five individuals in Family A, and on four individuals in Family B (Figure 1). The average coverage depth was 256.1×, with 98.8% of target bases covered by at least five reads. This supports a high level of confidence in the variant calling. As a result of the filtering procedure, three rare missense single-nucleotide variants in family with sequence similarity 65 member A (*FAM65A*), nuclear factor of activated T-cells 3 (*NFATC3*) and cadherin-related 23 (*CDH23*) were shown to be co-segregated in Family A and Family B in an autosomal-dominant manner.

### Variant in the 83 sporadic patients

In 83 Japanese and Korean sporadic patients of SMIA, 33 patients had variants in the reported causal gene (Table 1). *PAX9* variants (c.398A>G (*n*=1)), *AXIN2* variants (c.1250C>T (*n*=4), c.1807G>C (*n*=6), c.1853A>G (*n*=1) and c.2213C>T (*n*=2)), *EDA* variants (c.428G>C (*n*=1), c.436G>T (*n*=1) and c.555T>A (*n*=1)), *EDAR* variants (c.319A>G (*n*=10), c.701G>T (*n*=1), c.1138A>C (*n*=2), c.1166G>C (*n*=1), c.1270G>A (*n*=1)), *WNT10A* variants (c.511C>T (*n*=1), c.637G>A (*n*=4), c.874A>G (*n*=3), c.909C>A (*n*=1)) and *BMP2* variant (c.389G>A (*n*=1)). No variant was identified in *MSX1*, *EDARADD* or *GREM2*.

In 83 Japanese and Korean sporadic patients of SMIA, 38 patients have variants in *FAM65*, *NFATC3* and *CDH23* (Table 2). *FAM65A* variants (c.137C>T (*n*=5), c.148C>T (*n*=2), c.1117C>A (*n*=1), c.1994C>T (*n*=1), c.2542G>A (*n*=1), c.2668C>T (*n*=4), c.2734C>A (*n*=1) and c.2771G>A (*n*=5)), *NFATC3* variants (c.244A>G (*n*=4), c.281A>C (*n*=1), c.1043C>T (*n*=1) and c.1279C>G (*n*=1)) and *CDH23* variants (c.127G>A (*n*=9), c.141T>G (*n*=1), c.1637G>C (*n*=1), c.1681T>G (*n*=2), c.3179G>A (*n*=1), c.3352G>A (*n*=2), c.3397G>A (*n*=1), c.3656G>A (*n*=1), c.4009G>A (*n*=1), c.4135C>T (*n*=1), c.4346G>A (*n*=1), c.4762C>T (*n*=2), c.5722G>A (*n*=1) and c.6023G>T (*n*=1)).

Table 3 presented variants of candidate genes and scores of pathogenicity prediction tools.

## Discussion

In the present study, we attempted to identify genetic causalities of SMIA, which is prevalent in Asian populations. Two family with SMIA was extensively analyzed by exome sequencing and co-segregating genes were screened in 83 sporadic patients of Japanese and Koreans. Exome analyses identified variants in the reported genes for tooth agenesis, including *PAX9*, *AXIN2*, *EDA*, *EDAR*, *WNT10A* and *BMP2*. In addition, newly identified genes, including *FAM65*, *NFATC3* and *CDH23*, are reported as a possible causality.

Cadherin-23 is an atypical cadherin implicated in several deafness syndromes, including Usher syndrome, because of its role as a component of tip link structural proteins connecting sensory hairs of hair cells in the inner ear. *CDH23* variants are thought to impair the structural maintenance of the tip link and to reduce contact between sensory hairs, resulting in nonsyndromic hearing loss.^23^ The functional role of *FAM65A* has been unknown. The product of *NFATC3* is a member of the nuclear factors of activated T cells DNA-binding transcription complex. It is known to have a role in the regulation of gene expression in T cells and immature thymocytes.^24^

Experimental approaches in humans to identify genes that act on tooth development are limited. Therefore, the identification of genetic factors involved in defects of dentition such as tooth agenesis provides valuable information. Previous studies have shown that variants of *MSX1* phenotypically lead to tooth agenesis mostly at the third molars, second premolars and maxillary first premolars, while *PAX9* variants cause nonsyndromic tooth agenesis that preferentially affects the third molars, maxillary first and second molars and the mandibular second molars.^25^ Thus posterior teeth appear to be particularly sensitive to defects in *MSX1* and PAX9.^10^ Conversely, autosomal-dominant variants of *AXIN2* were shown to cause a severe form of tooth agenesis that preferentially affects the permanent molars, lower incisors and upper lateral incisors,^26^ while variants of *EDA* can lead to both hypohidrotic ectodermal dysplasia and nonsyndromic tooth agenesis favoring the anterior dentition.^27^ Thus the differential sensitivities of specific dentition types might depend on the differential expression of related genes and possibly reflect different roles for such genes during normal tooth development.^10,25^

Mouse teeth differ greatly from those of humans.^28^ For example, the labial side of the mouse incisor is coated with enamel and resembles a tooth crown, while the lingual side is more similar to a tooth root.^29^ Despite these differences in dentition, early stages of tooth development in both species are very similar, and the basis of tooth development and its molecular mechanisms originally discovered in the mouse have since been confirmed in humans.^23,25,27^ Knowledge from animal models, especially the mouse, is therefore important for our understanding of the genetic basis of tooth agenesis.^30^ Thus observation of *Cdh23* mRNA expression in mouse mandibular incisors is also expected to occur in humans (Supplementary Figures S1A,B and S2I). We showed that *Cdh23* is expressed in the central parts of the tooth buds and enamel organs and in the oral epithelia adjacent to the tooth germs only during the late bell stage (E16.5); it is not expressed in the maxillary incisors or mandibular molars in any stages (Supplementary Figure S2A–H). *Bmp2* is expressed in the lower incisors only during the bell stage, although it is also expressed in the lower molars throughout the initiation stage, bud stage, cap stage and bell stage.^31^ Odontoblasts differentiate from the dental papilla and produce the dentin matrix during the bell stage, and ameloblasts simultaneously arise from the epithelium and secrete the enamel matrix.^32^ *BMP2* reportedly has single-nucleotide polymorphisms that induce an increased risk of mandibular incisor agenesis.^11^

In summary, we found that novel variants in previously reported causal genes appear to mainly contribute to tooth agenesis of the anterior region, and the research using exome sequencing suggested the association between *FAM65A*, *NFATC3* and *CDH23* or SMIA. The early molecular diagnosis of tooth agenesis may enable to improve patient care^29^ and to alert clinicians to counsel patients to have regular colonoscopies in early diagnosis of *AXIN2* mutations.^30^ Moreover, the identification of causality for agenesis of the mandibular incisors, referred to herein as SMIA, may enable us to elucidate the precise molecular mechanisms of spatial gene regulation in the distinctive development of human dentition. In this study, some patients exhibited a genetic causality that could not be determined. The expression of >200 genes within teeth were studied, and a large number of candidates were given.^33^ A limitation of this study was the relatively small number of pedigrees; thus replication with a larger-scale study could narrow down the number of these candidate genes. Further studies are also required to determine the contribution of *FAM65A*, *NFATC3* and *CDH23* to the causality of SMIA in different populations and the agenesis of human teeth other than the mandibular incisors.

## Figures and Tables

**Table 1:** Number of variants of previous reported genes with a filtering methodology in 83 patients

*Gene*	*PAX9*	*AXIN2*				*EDA*			*EDAR*					*WNT10A*				*BMP2*
SH1																	c.909C>A	
SH2									c.319A>G									
SH3																		
SH4											c.1138A>C							
SH5			c.1807G>C															
SH6		c.1250C>T																
SH7																		
SH9															c.637G>A			
SH10									c.319A>G									c.389G>A
SH11															c.637G>A			
SH14				c.1853A>G												c.874A>G		
SH15							c.436G>T											
SH16									c.319A>G						c.637G>A			
SH18																		
SH19										c.701G>T								
SH21																		
SH23																		
SH25																		
SH26		c.1250C>T													c.637G>A			
SH28								c.555T>A										
SH29																c.874A>G		
SH31																		
SH32																		
SH33	c.398A>G																	
SH34																		
SH36		c.1250C>T																
SH37														c.511C>T				
SH38					c.2213C>T				c.319A>G									
SH39			c.1807G>C															
SH42																		
SH43			c.1807G>C		c.2213C>T													
SH44											c.1138A>C							
SH45																		
SH47																		
SH49									c.319A>G									
SH50																		
SH51																		
SH53																		
SH55																		
SH56																		
SH58																		
SH59																		
SH61																		
SH62									c.319A>G				c.1270G>A					
SH63																		
SH64																		
SH68									c.319A>G									
SH70			c.1807G>C													c.874A>G		
SH72									c.319A>G									
SH75						c.428G>C												
SH76																		
SH77		c.1250C>T							c.319A>G									
SH78												c.1166G>C						
SH79									c.319A>G									
SH80			c.1807G>C															
SH81																		
SH83			c.1807G>C															

Only individuals with any variants are shown. No variant was identified in *MSX1*, *EDARADD* or *GREM2*.

**Table 2:** Number of variants of FAM65A, NFATC3 and CDH23 gene

*Gene*	*FAM65A*								*NFATC3*				*CDH23*														
Affected famili A								c.2771G>A	c.244A>G															c.4762C>T			
Affected famili B								c.2771G>A	c.244A>G																c.5418C>G		
SH1		c.148C>T																									
SH2																											
SH3													c.127G>A														
SH4																											
SH5																											
SH6																											
SH7								c.2771G>A																		c.5722G>A	
SH9																											
SH10																											
SH11																							c.4346G>A				
SH14														c.141T>G													
SH15													c.127G>A														
SH16																											
SH18																c.1681T>G											
SH19													c.127G>A														
SH21													c.127G>A							c.3656G>A							
SH23																											c.6023G>T
SH25	c.137C>T																										
SH26		c.148C>T																									
SH28																											
SH29																											
SH31													c.127G>A														
SH32																		c.3352G>A									
SH33																c.1681T>G											
SH34																	c.3179G>A										
SH36																											
SH37																											
SH38																											
SH39																											
SH42								c.2771G>A	c.244A>G																		
SH43																											
SH44																											
SH45																								c.4762C>T			
SH47												c.1279C>G															
SH49																											
SH50						c.2668C>T																					
SH51													c.127G>A					c.3352G>A									
SH53				c.1994C>T																							
SH55	c.137C>T																										
SH56							c.2734C>A			c.281A>C	c.1043C>T		c.127G>A														
SH58	c.137C>T																										
SH59								c.2771G>A	c.244A>G																		
SH61					c.2542G>A																						
SH62																								c.4762C>T			
SH63						c.2668C>T		c.2771G>A	c.244A>G																		
SH64						c.2668C>T																					
SH68																					c.4009G>A						
SH70								c.2771G>A	c.244A>G																		
SH72																											
SH75			c.1117C>A																								
SH76	c.137C>T												c.127G>A														
SH77													c.127G>A														
SH78																											
SH79	c.137C>T					c.2668C>T																					
SH80															c.1637G>C							c.4135C>T					
SH81																			c.3397G>A								
SH83																											

Only individuals with any variants are shown.

**Table 3:** Identified variants and scores of pathogenicity prediction tools

*Gene*	*GenBank accession number*	*Substitution*		*Variant ID*	*MAF*		*PolyPhen2*	*GERP*
					*HGVD*	*1000 Genomes*		
*PAX9*	NM_006194	c.398A>G	p.Asn133Ser		0	0	0.985	4.90
*AXIN2*	NM_004655	c.1250C>T	p.Ala417Val		0.0141	0.0032	0.001	−4.86
	NM_004655	c.1807G>C	p.Ala603Pro	rs145353986	0.0492	0.0100	0.000	−2.26
	NM_004655	c.1853A>G	p.Asp618Gly		0	0	0.028	3.95
	NM_004655	c.2213C>T	p.Ser738Phe	rs139209450	0.0150	0.0032	0.004	1.02
*EDA*	NM_001005613	c.428G>C	p.Gly143Ala	rs61761321	0	0	0.364	4.82
	NM_001005613	c.436G>T	p.Gly146Cys		0	0	Unknown	0.00
	NM_001005609	c.555T>A	p.Asn185Lys		0	0	0.616	2.59
*EDAR*	NM_022336	c.319A>G	p.Met107Val	rs61761321	0.0485	0.0200	0.364	4.82
	NM_022336	c.701G>T	p.Ser234Ile		0	0	0.201	1.23
	NM_022336	c.1138A>C	p.Ser380Arg	rs146567337	0.0282	0.0100	0.978	5.18
	NM_022336	c.1166G>C	p.Gly389Ala		0	0	0.888	4.91
	NM_022336	c.1270G>A	p.Val424Met		0	0	0.888	4.91
*WNT10A*	NM_025216	c.511C>T	p.Arg171Cys	rs116998555	0.0158	0.0046	0.999	1.57
	NM_025216	c.637G>A	p.Gly213Ser	rs147680216	0.0156	0.0046	0.988	4.41
	NM_025216	c.874A>G	p.Ser292Gly		0.0040	0	0.001	2.25
	NM_025216	c.909C>A	p.H303Gln		0.0132	0	0.999	3.93
*BMP2*	NM_001200	c.389G>A	p.R130Gln		0.0012	0	0.023	5.70
*FAM65A*	NM_001193522	c.137C>T	p.Pro46Leu		0.0068	0.0009	0.793	4.82
	NM_001193522	c.148C>T	p.Pro50Ser	rs140402307	0.0037	0.0009	0.679	4.82
	NM_001193522	c.1117C>A	p.Leu373Met		0.0014	0.0009	0.009	1.17
	NM_001193522	c.1994C>T	p.Thr665Ile	rs28364801	0.0167	0.0018	0.212	2.30
	NM_001193522	c.2542G>A	p.Glu848Lys		0	0	0.676	4.91
	NM_001193522	c.2668C>T	p.Arg890Cys	rs61744916	0.0107	0.0600	0.038	1.96
	NM_001193522	c.2734C>A	p.Arg912Ser		0	0	0.026	1.18
	NM_001193522	c.2771G>A	p.Arg924His	rs145721165	0.0268	0.0032	0.983	5.15
*NFATC3*	NM_004555	c.244A>G	p.Ser82Gly	rs56326642	0.0215	0.0014	0.000	1.65
	NM_004555	c.281A>C	p.Glu94Ala	rs3743736	0.0156	0.0018	0.963	5.57
	NM_004555	c.1043C>T	p.Ala348Val	rs146091507	0.0156	0.0018	0.001	5.25
	NM_004555	c.1279C>G	p.His427Asp		0	0	0.996	5.13
*CDH23*	NM_001171933	c.127G>A	p.Val43Ile	rs41281334	0.0248	0.0400	0.014	3.21
	NM_001171933	c.141T>G	p.Asn47Lys		0	0	1	5.49
	NM_001171930	c.1637G>C	p.Arg546Pro		0	0	0.976	0.49
	NM_001171933	c.1681T>G	p.Phe561Val	rs3802707	0.0175	0.0014	0.011	3.09
	NM_001171930	c.3179G>A	p.Arg1060Gln		0	0	1.000	5.49
	NM_001171930	c.3352G>A	p.Gly1118Ser		0.0055	0	0.272	4.92
	NM_001171930	c.3397G>A	p.Glu1133Lys		0	0	0.668	4.89
	NM_001171930	c.3656G>A	p.Arg1219Gln		0	0	1.000	4.32
	NM_001171930	c.4009G>A	p.Ala1337Thr		0	0	1.000	4.81
	NM_022124	c.4135C>T	p.Arg1379Cys		0	0	0.028	0.91
	NM_022124	c.4346G>A	p.Gly1449Asp		0	0	0.045	1.01
	NM_022124	c.4762C>T	p.Arg1588Trp	rs137937502	0.0051	0.0018	1.000	3.24
	NM_022124	c.5418C>G	p.Asp1806Glu	rs74145660	0.0918	0.0300	0.998	−5.31
	NM_022124	c.5722G>A	p.Val1908Ile		0.0040	0	0.028	−0.78
	NM_022124	c.6023G>T	p.Gly2008Val		0	0	0.045	1.01

Abbreviations: GERP, Genomic Evolutionary Rate Profiling; HGVD, Human Genetic Variation database; MAF, minor allele frequency (based on 1000 Genomes allele frequencies for Japanese and European populations); PolyPhen2, Polymorphism Phenotyping v2.
