# Supplementary material for: Comprehensive genetic exploration of selective tooth agenesis of mandibular incisors by exome sequencing
Source: Hum Genome Var. 2017 Feb 23;4:17005–. doi: 10.1038/hgv.2017.5 (PMC5321669; doi:10.1038/hgv.2017.5)

Comprehensive genetic exploration of selective tooth agenesis of mandibular incisors by exome sequencing

Tetsutaro Yamaguchi[1]*, Kazuyoshi Hosomichi[2,3]*, Keisuke Yano[4], Yong-Il Kim[5], Hirofumi Nakaoka[2], Ryosuke Kimura[6], Hirotada Otsuka[7], Naoko Nonaka[7], Shugo Haga[1], Masahiro Takahashi[1], Tatsuo Shirota[8], Yoshiaki Kikkawa[9], Atsushi Yamada[10], Ryutaro Kamijo[10], Soo-Byung Park[5], Masanori Nakamura[7], Koutaro Maki[1], Ituro Inoue[2]

[1]Department of Orthodontics, School of Dentistry, Showa University, Tokyo, Japan; [2]Division of Human Genetics, National Institute of Genetics, Shizuoka, Japan; [3]Department of Bioinformatics and Genomics, Graduate School of Medical Sciences, Kanazawa University, Kanazawa, Ishikawa, Japan; [4]Verde Orthodontic Dental Clinic, Tokyo, Japan; [5]Department of Orthodontics, School of Dentistry, Biomedical Research Institute, Pusan National University Hospital, Busan, South Korea; [6]Department of Human Biology and Anatomy, Graduate School of Medicine, University of the Ryukyus, Okinawa, Japan; [7]Department of Oral Anatomy and Developmental Biology, Showa University School of Dentistry, Tokyo, Japa; [8]Department of Oral and Maxillofacial Surgery, School of Dentistry, Showa University, Tokyo, Japan; [9]Mammalian Genetics Project, Tokyo Metropolitan Institute of Medical Science, Tokyo, Japan; [10]Department of Biochemistry, School of Dentistry, Showa University, Tokyo, Japan; *These authors contributed equally to this work

Correspondence: Ituro Inoue (itinoue@nig.ac.jp) and Tetsutaro Yamaguchi (tyamaguchi@dent.showa-u.ac.jp)

Supplementary Figure 1A: In situ hybridization (ISH) and immunohistochemistry (IHC) analyses of Cdh23 expression during mouse tooth development. In A, ISH is shown on the left, and IHC on the right. Cross-sectional ISH and IHC demonstrating Cdh23 expression in the incisor germs at E16.5.

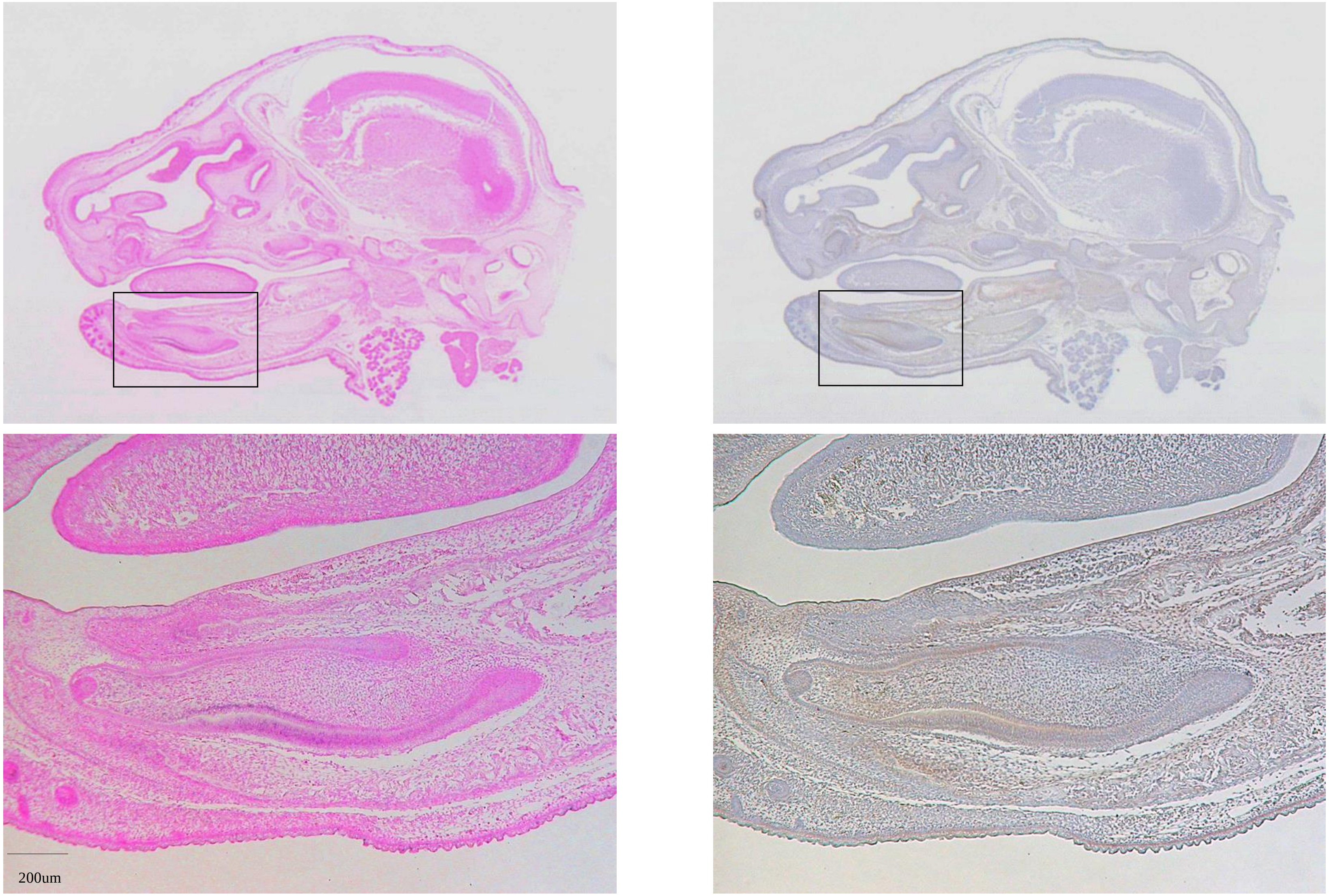

Supplementary Figure 1B: The boxed area in A is shown at higher magnification in B, focusing on mandibular incisors. In B, ISH is shown at the top, and IHC at the bottom. Cdh23 was expressed in morphologically distinct ameloblasts and odontoblasts.

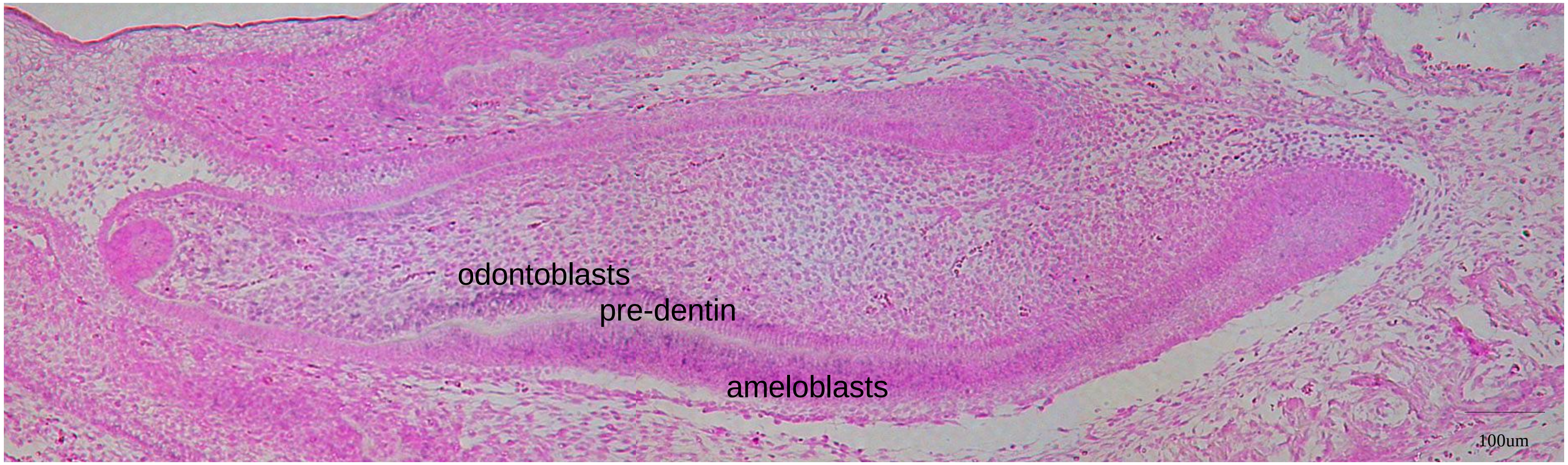

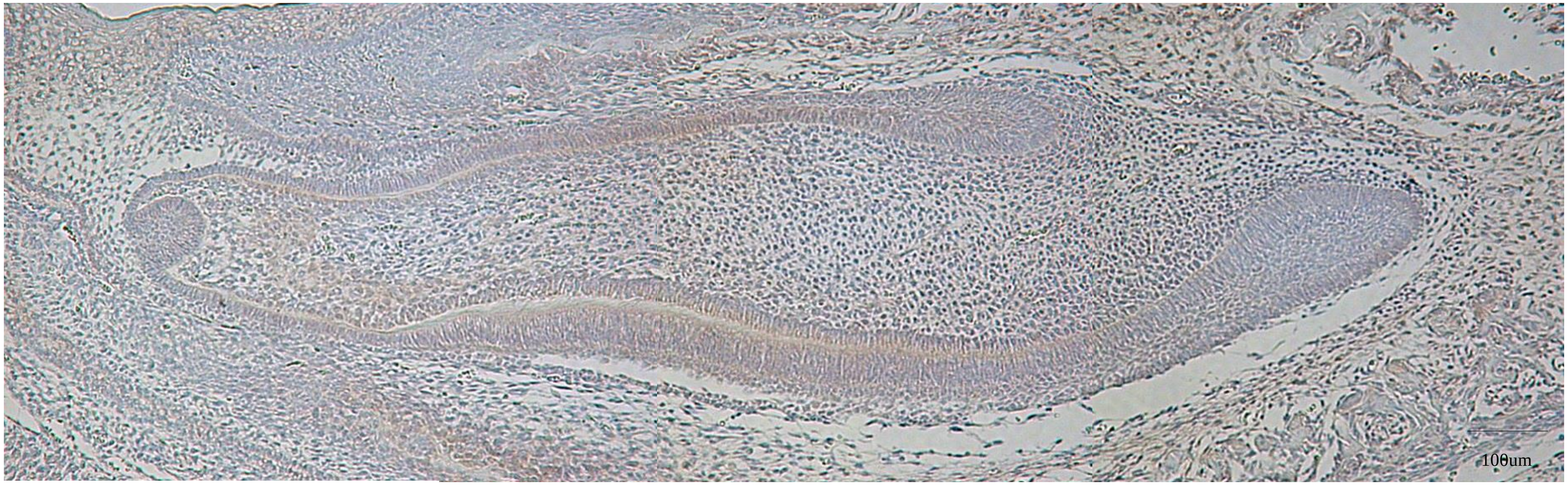

Supplementary Figure 2A: ISH and IHC on frontal section showing Cdh23 expression at E12.5 in mandibular molars.

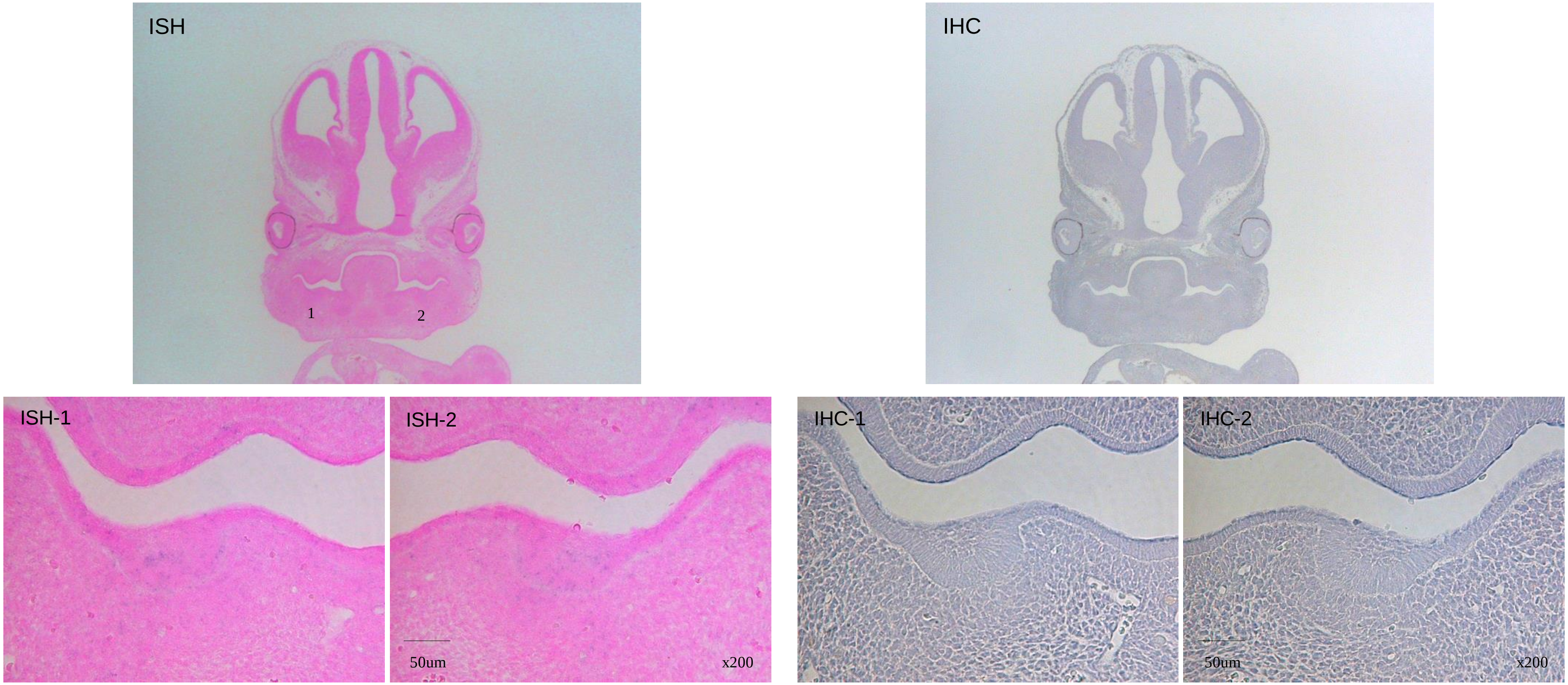

Supplementary Figure 2B: ISH and IHC on frontal section showing Cdh23 expression at E12.5 in maxillary incisors.

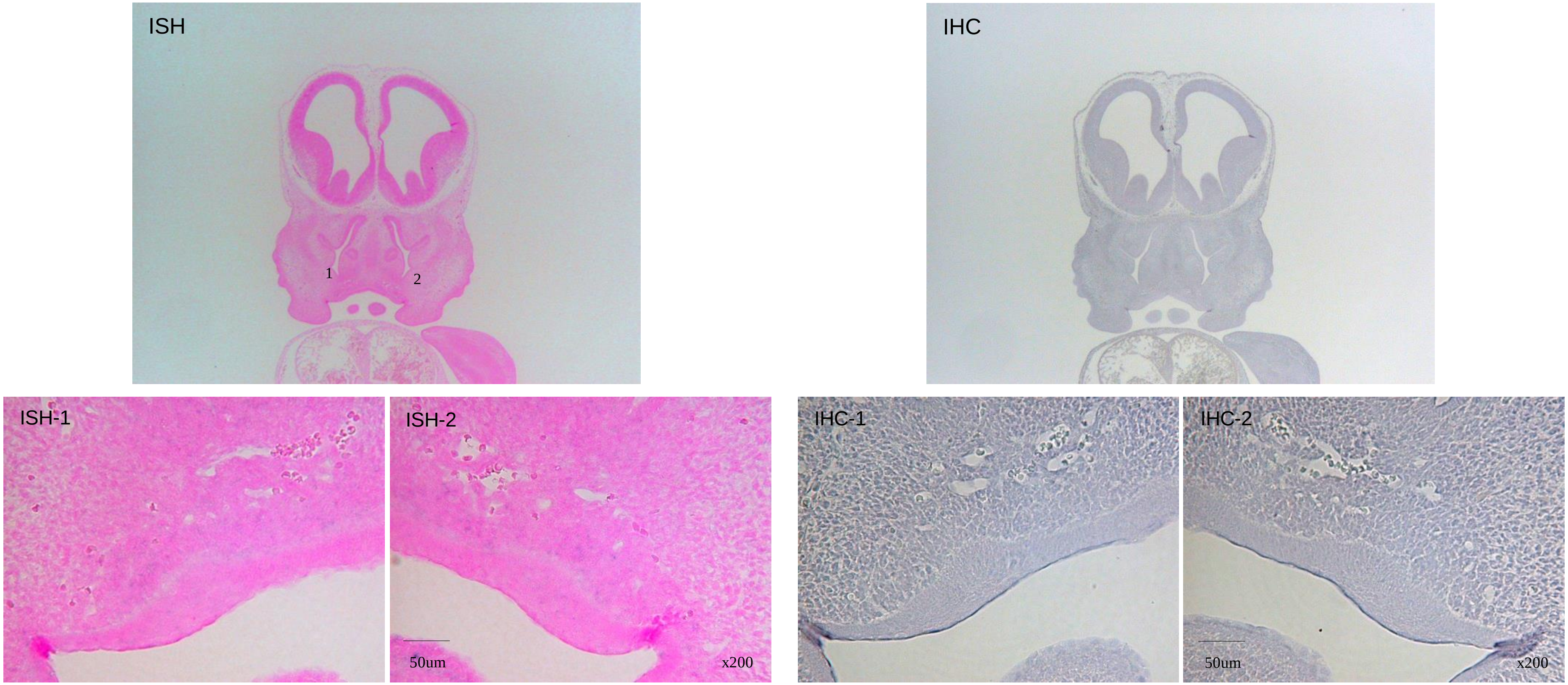

Supplementary Figure 2C: ISH and IHC on frontal section showing Cdh23 expression at E12.5 in mandibular incisors.

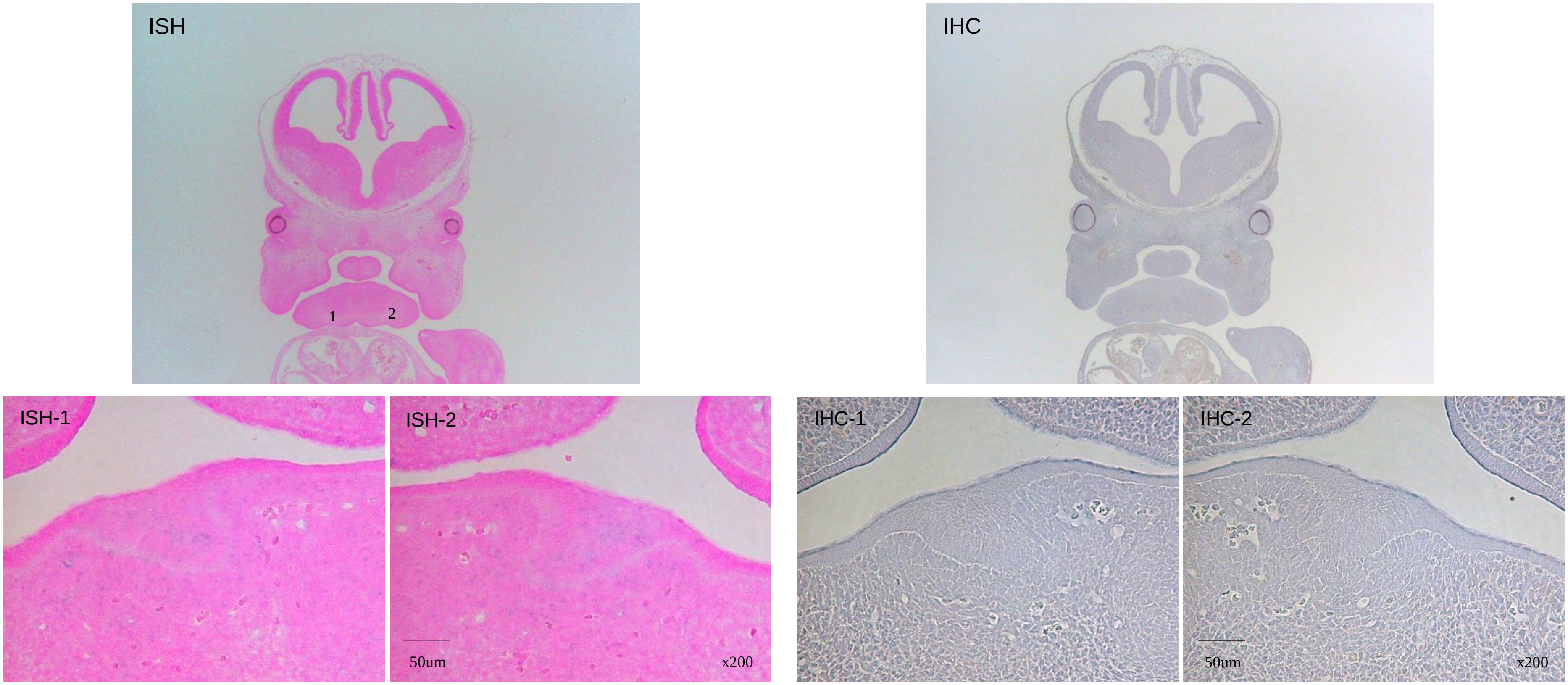

Supplementary Figure 2D: ISH and IHC on frontal section showing Cdh23 expression at E14.5 in mandibular molars.

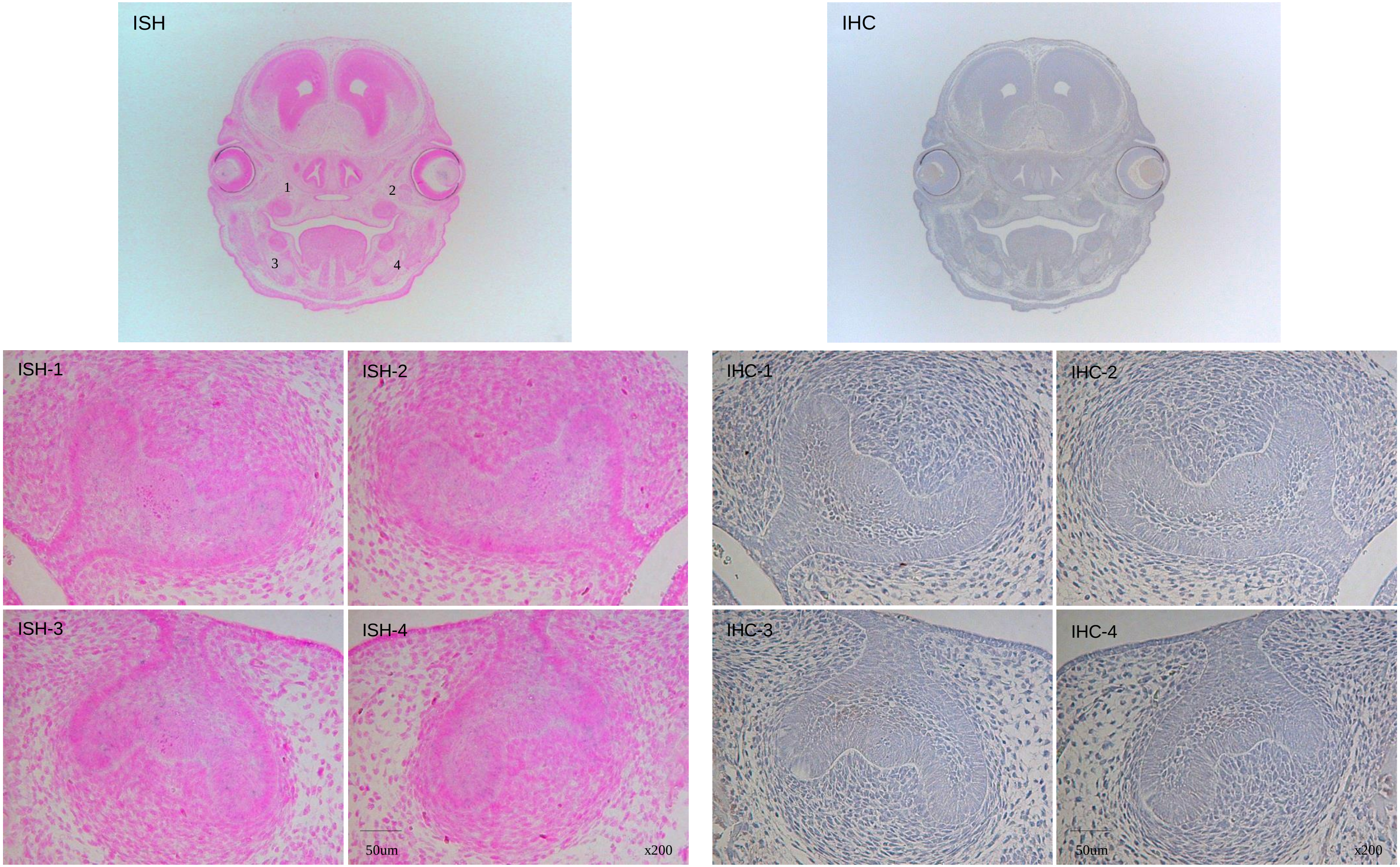

Supplementary Figure 2E: ISH and IHC on frontal section showing Cdh23 expression at E14.5 in maxillary incisors.

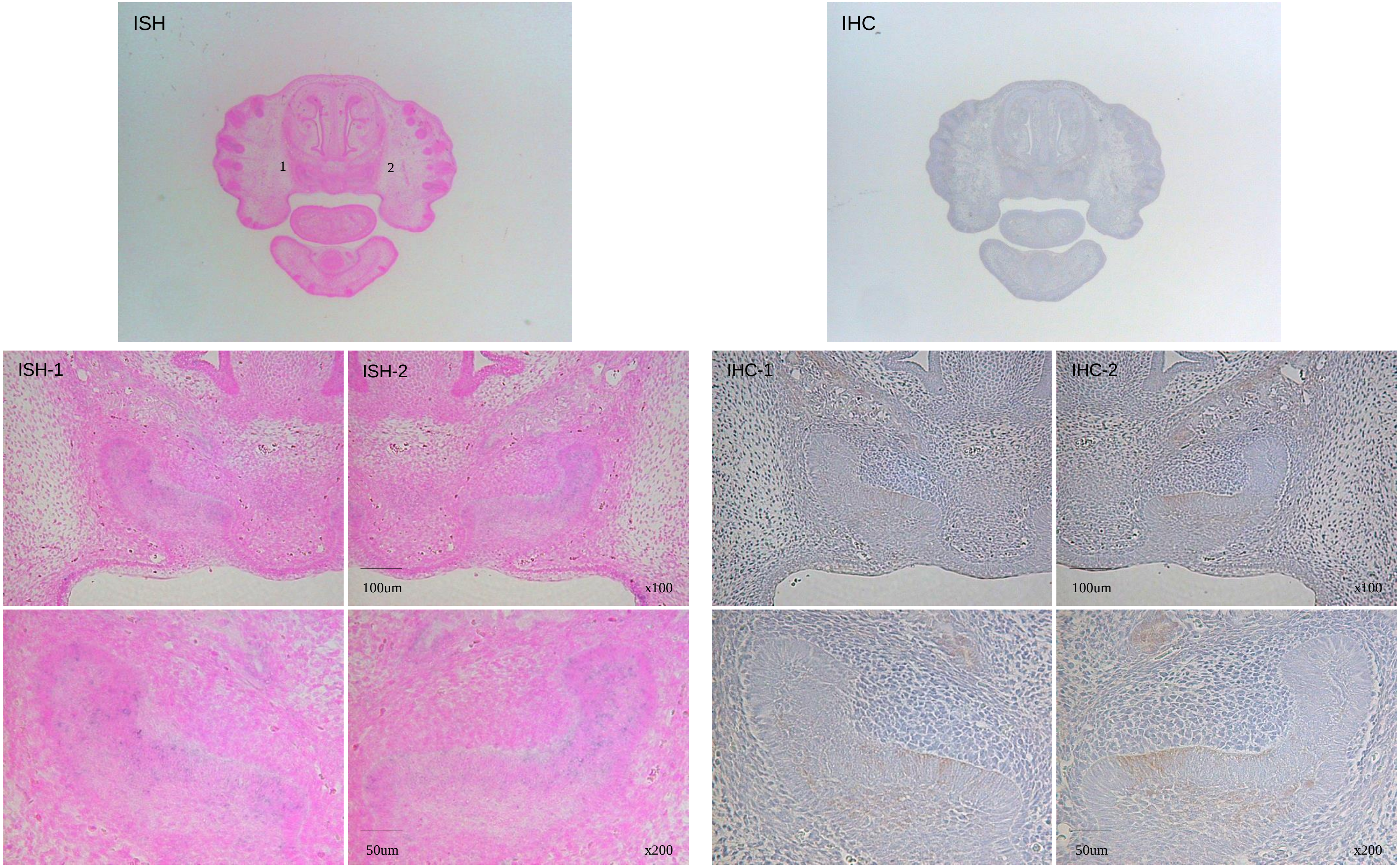

Supplementary Figure 2F: ISH and IHC on frontal section showing Cdh23 expression at E14.5 in mandibular incisors.

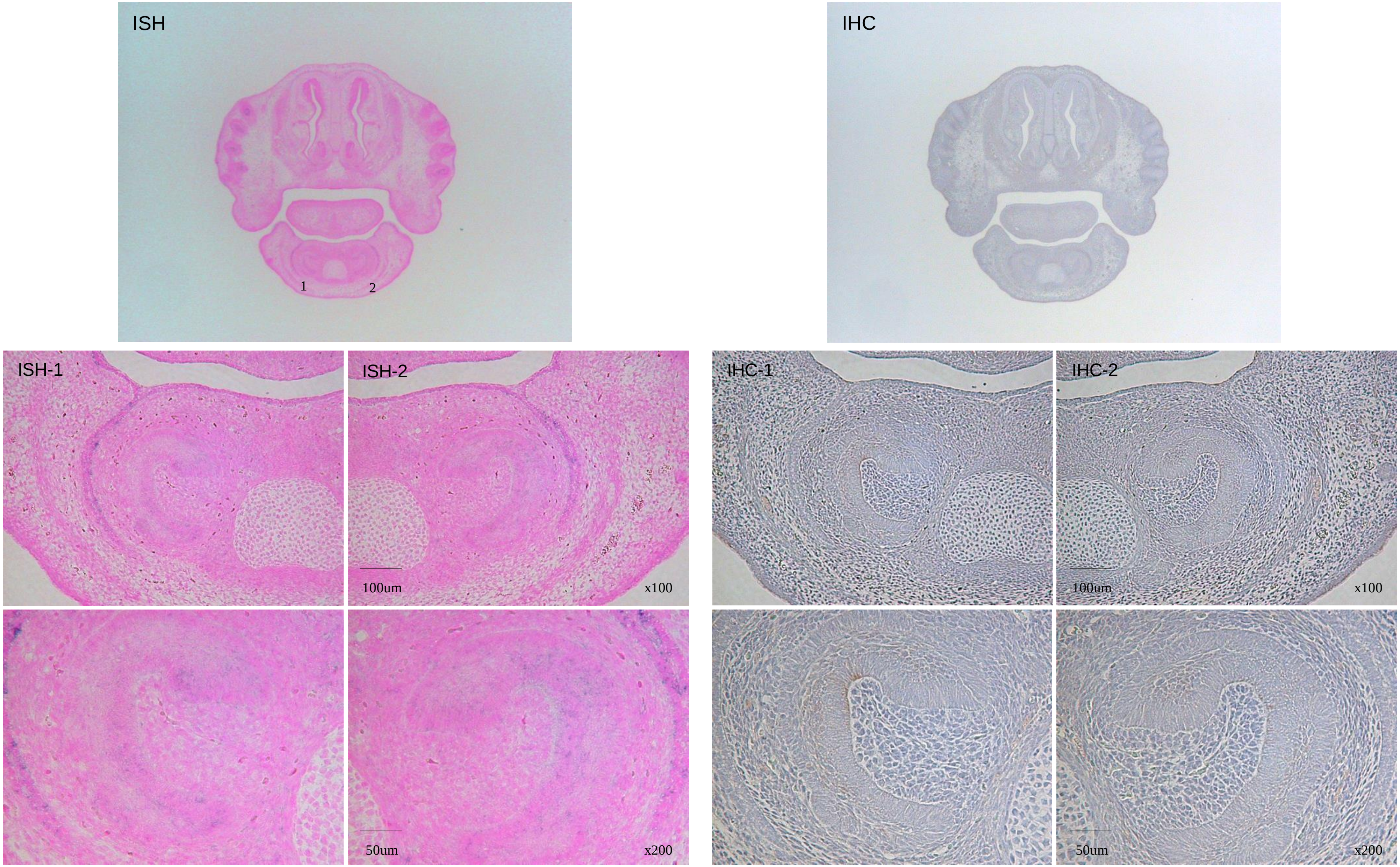

Supplementary Figure 2G: ISH and IHC on frontal section showing Cdh23 expression at E16.5 in mandibular molars.

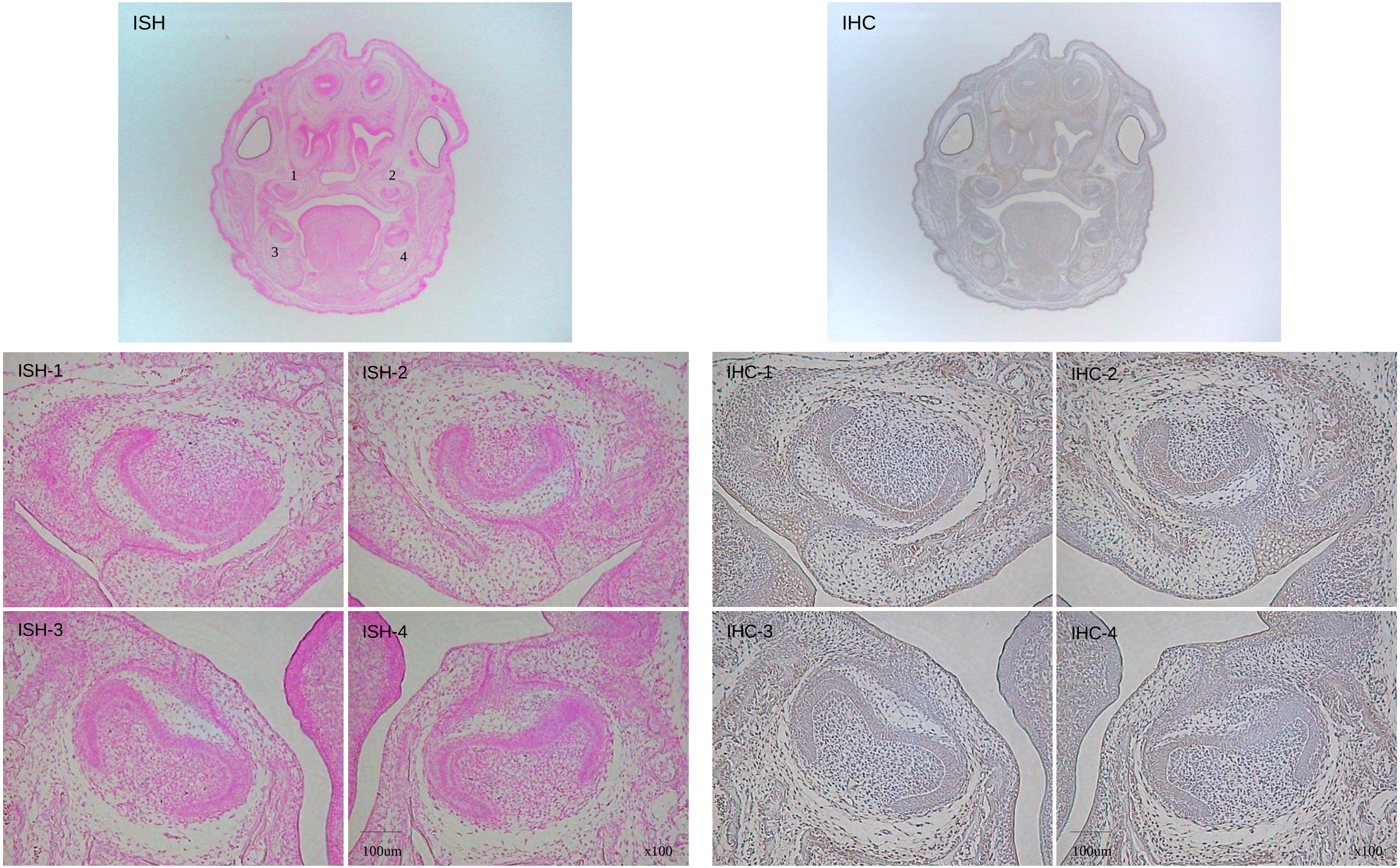

Supplementary Figure 2H: ISH and IHC on frontal section showing Cdh23 expression at E16.5 in maxillary incisors.

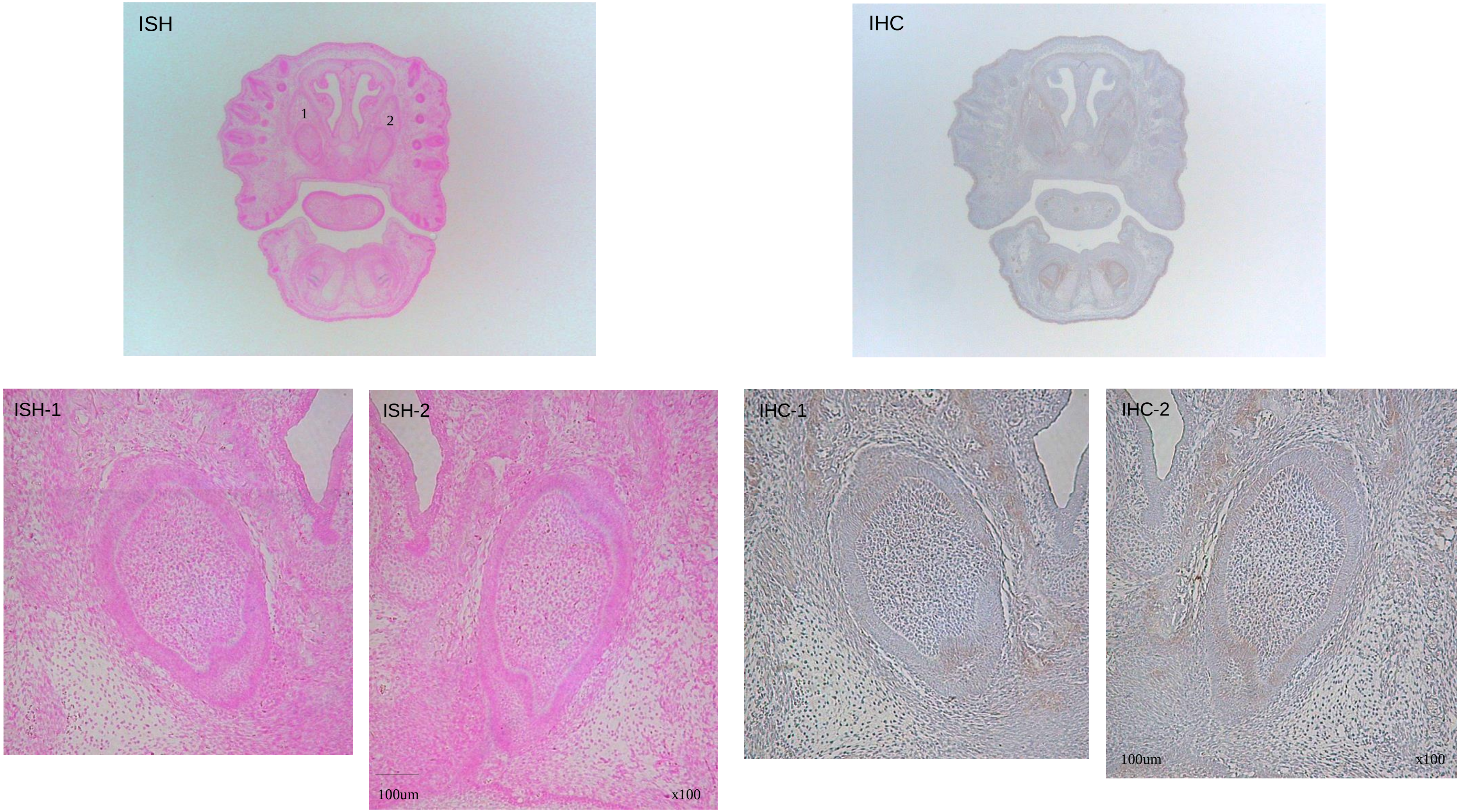

Supplementary Figure 2I: ISH and IHC on frontal section showing Cdh23 expression at E16.5 in mandibular incisors. Cdh23 was expressed in mandibular incisors at the late bell stage.

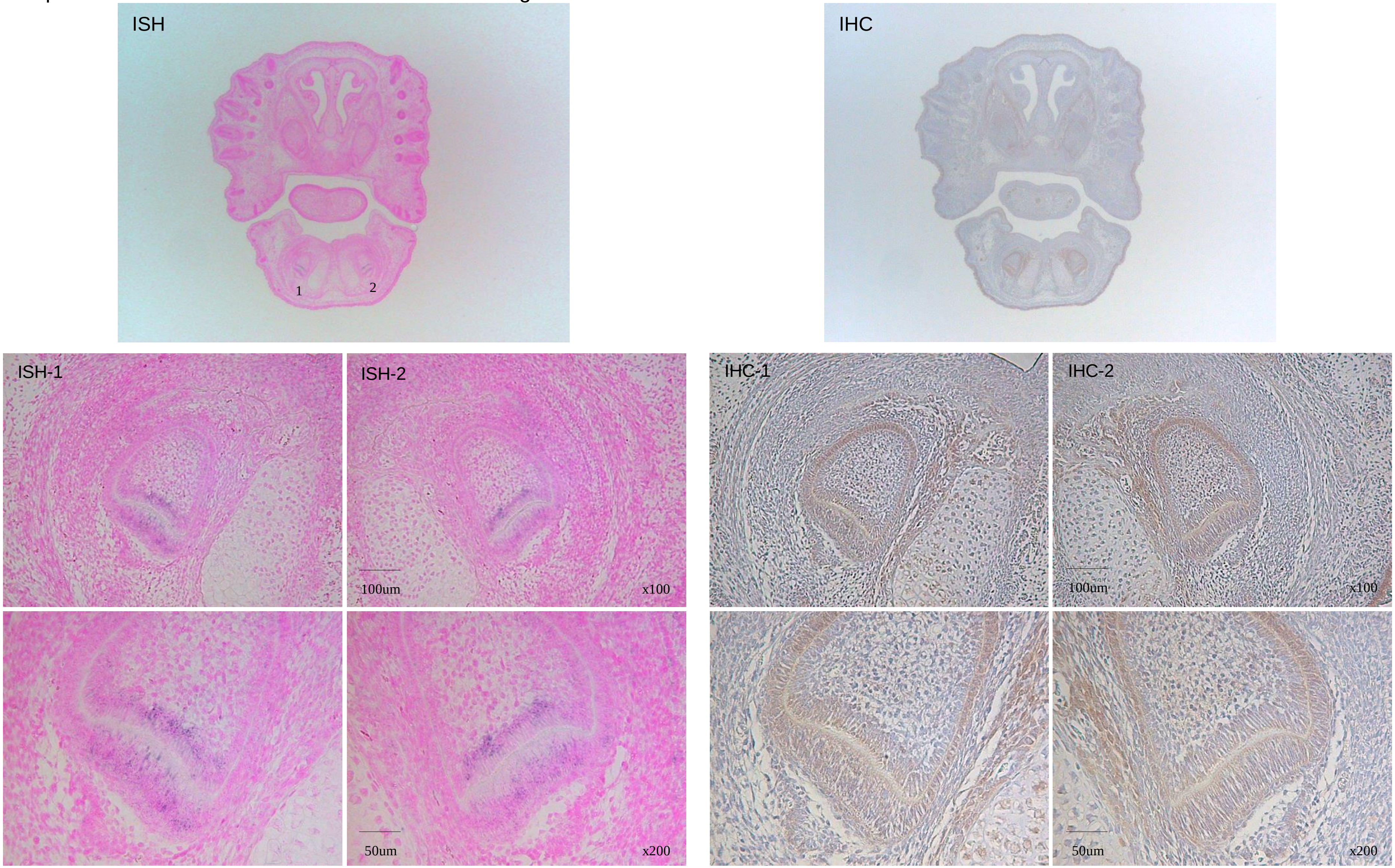

Supplement: Supplementary Figures [file hgv20175-s1.pdf]
